# Recognition of facial emotion based on SOAR model

**DOI:** 10.3389/fnins.2024.1374112

**Published:** 2024-05-17

**Authors:** Matin Ramzani Shahrestani, Sara Motamed, Mohammadreza Yamaghani

**Affiliations:** ^1^Department of Computer Engineering, Rasht Branch, Islamic Azad University, Rasht, Iran; ^2^Department of Computer Engineering, Fouman and Shaft Branch, Islamic Azad University, Fouman, Iran; ^3^Department of Computer Engineering, Lahijan Branch, Islamic Azad University, Lahijan, Iran

**Keywords:** facial emotional recognition, 3D convolutional neural network (3DCNN), learning automata (LA), SOAR model, deep learning

## Abstract

**Introduction:**

Expressing emotions play a special role in daily communication, and one of the most essential methods in detecting emotions is to detect facial emotional states. Therefore, one of the crucial aspects of the natural human–machine interaction is the recognition of facial expressions and the creation of feedback, according to the perceived emotion.

**Methods:**

To implement each part of this model, two main steps have been introduced. The first step is reading the video and converting it to images and preprocessing on them. The next step is to use the combination of 3D convolutional neural network (3DCNN) and learning automata (LA) to classify and detect the rate of facial emotional recognition. The reason for choosing 3DCNN in our model is that no dimension is removed from the images, and considering the temporal information in dynamic images leads to more efficient and better classification. In addition, the training of the 3DCNN network in calculating the backpropagation error is adjusted by LA so that both the efficiency of the proposed model is increased, and the working memory part of the SOAR model can be implemented.

**Results and discussion:**

Due to the importance of the topic, this article presents an efficient method for recognizing emotional states from facial images based on a mixed deep learning and cognitive model called SOAR. Among the objectives of the proposed model, it is possible to mention providing a model for learning the time order of frames in the movie and providing a model for better display of visual features, increasing the recognition rate. The accuracy of recognition rate of facial emotional states in the proposed model is 85.3%. To compare the effectiveness of the proposed model with other models, this model has been compared with competing models. By examining the results, we found that the proposed model has a better performance than other models.

## 1 Introduction

Facial expression recognition (FER) is an emerging research topic. FER uses computer technology and image processing to analyze and identify the types, intensity, and duration of facial expressions, and reveals a person’s emotional state by detecting subtle changes. Applications of FER in healthcare, security, safe driving, etc., have contributed to the validation of these methods and their adoption in human–computer interaction for intelligent results. Computational FER mimics human facial expression coding skills. Similarly, based on deep learning and artificial intelligence (AI) techniques, FER methods have been developed with edge modules to ensure efficiency and real-time processing. For this purpose, several studies have investigated different aspects of FER (Sajjad et al., 2023).

Emotions are an essential driver in decision-making and human communication. With the recent increase in human–computer interaction, emotional computing has become a popular research topic, aiming to develop computational systems that can understand and respond to human emotions (Leong et al., 2023). According to research, more than 90% of the concepts exchanged between people are transmitted through different channels, and facial expressions and body movements play a unique role in Mehrabian (2017). Emotion plays a vital role in communication. For example, frowning is a sign of dissatisfaction and agreement. Surprise, joy, and fear are natural human reactions to environmental factors.

Among the new studies in this field in the study mentioned in Han et al. (2024), a biomimetic model that imitates the function of the human brain and aligns with human perception and provides a better interpretation capability for biological characteristics is presented. Based on biological features, it has developed a non-linear neural network to extract sequential features, which is useful for dynamic emotion analysis. Or in the study mentioned in Liu et al. (2024), he used a Bayesian convolutional neural network with a chaotic multi-branch structure for this diagnosis. Aiming to address uncertainty issues, this model enables the network’s decisions to become more certain by increasing training accuracy.

Understanding facial expressions can help regulate emotional responses, such as enhancing a positive mood or reducing a negative emotional state, especially when the stimulating facial expression matches one’s intentions. In other words, it matches one’s monitoring efforts. Existing research studies generally use two types of classification: dynamic and static. Dynamic classifiers [such as Hidden Markov Model (HMM)] use multiple video frames and perform classification by analyzing temporal patterns from analyzed regions or extracted features. Static classifiers classify each frame in a video into a set of facial expressions based on the results of specific video frames. In general, these methods are such that some features of the images are extracted first. They are classified in a classification system, so one of the emotional categories is chosen. Automatic emotion recognition from face video images faced many challenges, including finding the face in the image, localizing the dimensions of the eyes, nose, and mouth, revealing the changes of the face and its components in a certain period, and establishing the relationship. It is very difficult to match these changes with one’s emotional expression. Every subject has its own variation depending on environmental and personal conditions. For example, when recognizing a face and finding the exact location of its components, the composition and appearance of the face, such as wearing glasses and the angle of the head, present several problems, each of which is the subject of extensive independent research for emotion recognition from video images (Arora et al., 2022; Dias et al., 2022; Li and Gao, 2022; Ma et al., 2022; Zhu et al., 2022; Alphonse et al., 2023; Höfling et al., 2023; Zhao et al., 2023).

Available scientific evidence suggests that people sometimes smile when they are happy, frown when they are sad, and frown when they are angry more often than would be expected by chance, as suggested by the popular view. However, the way people relate to anger, disgust, fear, joy, sadness, and surprise varies across cultures, situations, and even between individuals within the same situation. Furthermore, similar configurations of facial movements variably express instances of more than one emotion category. In fact, a certain configuration of facial movements, such as frowning, often conveys something other than an emotional state. Scientists agree that facial expressions convey a wide range of information and are important for social, emotional, or other communication. However, our review shows an urgent need for research that examines how people move to express emotions and other social information in the diverse contexts that make up everyday life, as well as a detailed study of the mechanisms by which people perceive instances of emotion.

The model presented in this article has two main features: A model for better representation of visual features and learning the time sequence of frames in video and introducing the new model for visual processes. For this purpose, 10 different frames are separated from the beginning of the video file. The frame difference is considered to be a change of more than three points from the last frame. This operation is done in this way, and all the extracted frames are divided by 10 and the first frame from each divided part will be selected as the selected frame. The reason for using this mode is to avoid selections at the end of the video file, which is mostly motionless. To better display the visual features, the combination of 3DCNN and LA has been used. This combination will increase learning and show better features. The procedure sequence of the proposed model is as follows: all the videos of the input data set are read, and its basic frames are extracted in the specified number. This article uses the SOAR model to improve the recognition rate and uses the cognitive science model compatible with the human brain. In the following section, features based on the improvement of deep learning are selected to reduce the dimensions and increase the efficiency of the proposed method. Therefore, 3DCNN-LA was used to choose visual features. The best features are extracted with high precision, and they reveal any unwanted stress or anxiety that can be hidden from monitoring. In addition, in the feature selection stage, the reason for choosing 3DCNN on face images is that no dimension is removed from the images. Considering the temporal information in dynamic images leads to more efficient and better feature selection. In the following, an improved SOAR model with a combination of 3DCNN and LA is used for classification. Learning automata (LA) has been used to improve the SOAR cognitive science model. LA can continuously learn throughout its life cycle, enhance learning with a reward mechanism in mind, and learn from close examples. The state symbolizes long-term and short-term working memory changes based on environmental changes. Therefore, the proposed LA method is used to improve the SOAR model in the learning section. The main reason for using LA in the proposed model is that this algorithm can improve its behavior using its previous experience. This algorithm’s first position and action are random, making subsequent moves based on the environmental response it receives.

The main parts of this article are organized in such a way that section 2 is related to the review of the studies done on the recognition of emotional states of the face. The following section presents the proposed improved SOAR method, and finally, the test results and conclusions are presented in the following sections.

## 2 Recognition of emotional states

### 2.1 Face emotion recognition

In the study mentioned in Höfling et al. (2023), we investigated the relationships between facial movements (i.e., action unit activity) and self-reported emotions of commercials, advertisements, and brand effects with automatic facial coding. In the study mentioned in Zhao et al. (2023), the authors proposed a global–local GAN based on stylistic attention to apply the features of a subject, to generate personalized caricatures. To integrate a subject’s facial features, they introduced a point-based warping controller for personalized shape exaggeration. It uses facial landmarks as control points to warp the image according to its facial features without the need for caricature facial landmarks. To properly integrate the face feature with the caricature style, they introduced the style attention module, which uses the attention mechanism instead of simple adaptive instance normalization (AdaIN) for style transfer. To reduce the wrong cases and increase the quality of the generated caricatures, they proposed a multi-scale discriminator for the global and local differentiation of the synthesized and accurate caricatures, improving the structure and actual details of the synthesized caricatures. In the study mentioned in Ma et al. (2022), to determine whether Chinese people show different recognition accuracy and gaze patterns for Asian (own race) and White (other race) facial expressions (neutral, happy, sad, angry, disgust, and fear) or not, 89 healthy Chinese adults viewed Asian and White facial expressions during eye tracking and were subsequently required to identify the expressions and rate their intensity and effect on arousal. The results showed that subjects recognized sad expressions in Asian faces better than White faces. On the other hand, recognition accuracy was higher for White neutral, happy, fearful, and disgusted expressions, although this may be because subjects often misclassified these Asian expressions as sad. In the study mentioned in Zhu et al. (2022), the relationship between facial emotion recognition and psychological characteristics is first discussed. Based on this, a face emotion recognition model is built by adding convolutional neural network (CNN) layers and integrating CNN with several neural networks, such as VGGNet, AlexNet, and LeNet-5. Second, based on feature fusion, a central local binary (CLBP) algorithm optimized to CNN is introduced to construct a CNN-CLBP algorithm for facial emotion recognition. Finally, the validity analysis is performed on the algorithm after preprocessing the face images and optimizing the relevant parameters. Compared with other methods, the CNN-CLBP algorithm has a higher accuracy in recognizing facial expressions, with an average recognition rate of 88.16%. In addition, the detection accuracy of this algorithm is improved by image preprocessing and parameter optimization, and there is no poor fit. In addition, the CNN-CLBP algorithm can recognize 97% of happy and surprised expressions, but the misidentification rate of sad expressions is 22.54%. The research results are the data reference and orientation for analyzing the psychological–logical characteristics of teenagers involved in crimes. In the study mentioned in Li and Gao (2022), a face recognition model is built based on a hybrid network of multi-scale features, aiming to make full use of face features and improve face recognition accuracy. In addition, three different scale networks are designed to extract the global features of faces. In the study mentioned in Arora et al. (2022), the authors improved the convolutional neural network technique to detect seven basic emotions and evaluated several preprocessing methods to show how they affect CNN performance. This research focuses on enhancing facial features and expressions based on emotional recognition. By identifying or recognizing facial expressions that evoke human responses, computers can make more accurate predictions about people’s mental states and provide more tailored responses. In the study mentioned in Alphonse et al. (2023), a new hybrid neural network (NN) model is proposed, which is capable of recognizing in real-time applications. Several NN models are compared for the first time in this study. Then, a hybrid NN model is created by combining a convolutional neural network (CNN), a recurrent neural network [RNN, i.e., long short-term memory (LSTM)], and a vision transformer. In the study mentioned in Dias et al. (2022), the authors proposed a facial emotion recognition method for masked face images using low-light image enhancement and feature analysis of upper facial features with a convolutional neural network.

### 2.2 Learning automata

**Figure 1:**
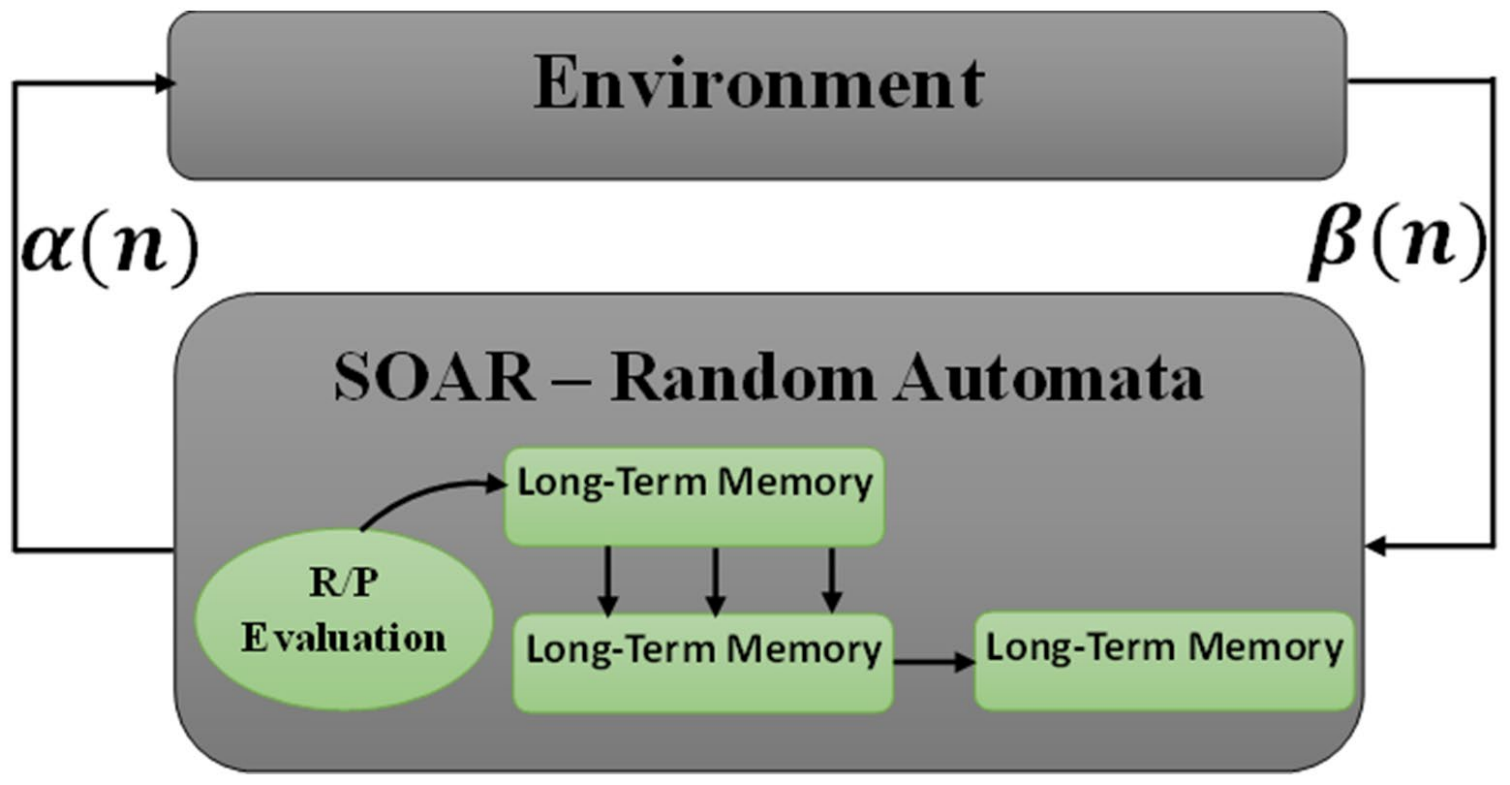
A view of the interaction between the environment and the automatic learning of automata.

As one of the techniques of artificial intelligence, the LA is a stochastic model that works in the reinforcement learning framework. Automatic learning inputs the selected action to its stochastic and operational environment. Reaching the ideal answer, the environment uses reinforcement feedback to respond to the approach of the adopted action to the ideal goal. The action probability vector is updated using the reinforcement feedback. Automatic learning tries to find the optimal action from the set of actions to minimize the average penalty of the environment. Automatic learning can be useful in systems where complete information about the environment is unavailable (Wongthanavasu and Ponkaew, 2016; Tan et al., 2021). The LA is classified into two main categories: fixed and variable structures. Variable structure learning automata are represented by the triple {β, α, T}, where β is the set of inputs, α is the set of actions, and T is the learning algorithm. The learning algorithm is used to modify the action probability vector as an iterative relation. Figure 1 shows an interaction between the environment and the considered automatic learning.

Let a _i_( n ) ∈ a and P ( n ) represent the action the learning automaton selects and the probability vector defined on the action set at the moment n, respectively. Let a and b denote the reward and penalty parameters, and let the penalty determine the increase and decrease of action probabilities, respectively. In addition, consider r as the number of actions that may be performed by machine learning. At every n moment, the action probability vector P ( n ) is updated by the linear learning algorithm presented in equation (1) if the environment rewards the chosen action a _i_( n ). If the action is penalized, it will be updated according to equation (2).


(1) $$\{\begin{array}{c} P_{i}\left(n+1\right)=P_{i}\left(n\right)+a\left[1-P_{i}\left(n\right)\right]\;j=i\\ P_{j}\left(n+1\right)=\left(1-a\right)P_{j}\left(n\right)\forall j\neq i \end{array}$$

(2) $$\{\begin{array}{c} P_{i}\left(n+1\right)=\left(1-b\right)P_{i}\left(n\right)\;j=i\\ P_{j}\left(n+1\right)=\frac{b}{r-1}+\left(1-b\right)P_{j}\left(n\right)\forall j\neq i \end{array}$$

Repetition 1 and 2 are called linear reward-penalty (LR-P) algorithms if a = b , and the given equations are called linear-epsilon reward penalty (LR − εP), where a > b . Finally, they are called linear reward-inaction (LR − I), where b=0 . If the learning automaton chooses an action like $\alpha_{i}$ in the nth repetition and receives a favorable response from the environment, $p_{i}$ (n; probability of action $\;\alpha_{i}$ ) increases and the probability of other actions decreases. On the contrary, if the response received from the environment is unfavorable, the probability of action $\alpha_{i}$ decreases and the probability of other automata actions increases. In any case, changes are made in such a way that the sum of $p_{i}$ (n) always remains constant and equal to one.

## 3 The proposed model

**Figure 2:**
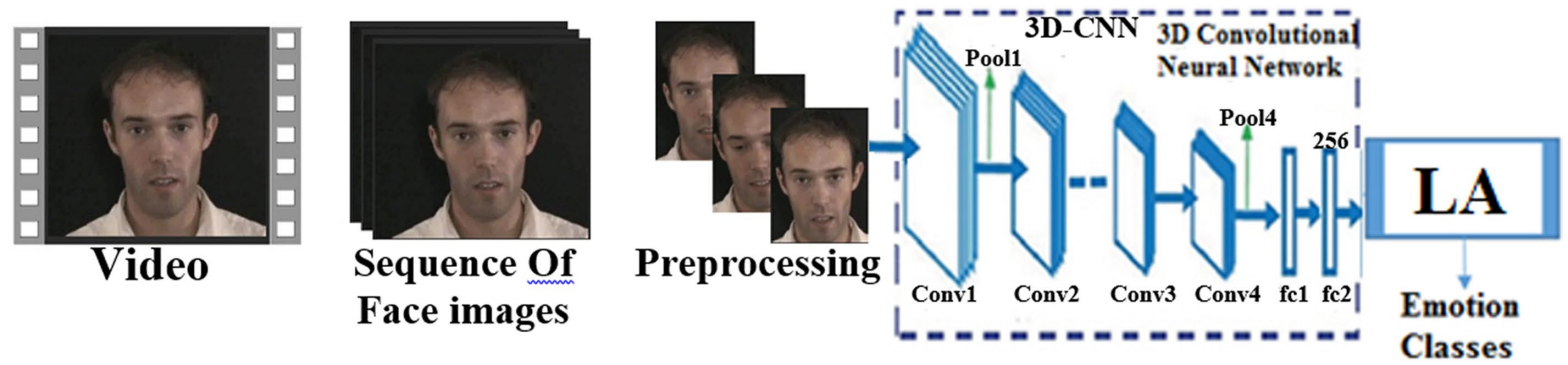
Block diagram of the proposed method. Facial images reproduced from the eNTERFACE’05 EMOTION Database under the terms of the MIT License (https://enterface.net/enterface05/main.php?frame=emotion).

In this section, the proposed model is explained, providing details of the substructure of each component of it. Figure 2 shows the block diagram of the proposed method.

According to Figure 2, the first step is to read the videos from the interface 05 datasets. To extract the image frames from the video, the separation algorithm with a specific frame rate from the video will be applied to all the samples. Face images are stored in particular folders, and then pre-processing operations are applied to the frames of images from the video. Video samples are divided into three categories: training, validation, and testing. For this purpose, 10 different frames are separated from the beginning of the video file. The frame difference is considered to be a change of more than three points from the last frame. First of all, 10 different frames are separated from the beginning of the video file. The frame difference is considered to be a change of more than three points from the last frame. This operation is done in this way, all the extracted frames are divided by 10 and the first frame from each divided part will be selected as the selected frame. The reason for using this mode is to avoid selections at the end of the video file, which is mostly motionless. To better display the visual features, the combination of 3DCNN and LA has been used. This combination will increase learning and show better features. In the end, the image frames of each video sample are sent to the 3DCNN network in the form of a three-dimensional matrix, including the number of sequences of frames of each video, width, height, and the number of image channels. The reason for selecting 3DCNN is that this method can store data in the entire network, deal with incomplete knowledge, and tolerate high errors. Therefore, it is expected to select appropriate features. Finally, a combination of 3DCNN and LA described has been used to classify the obtained features and simulate the SOAR model. In addition, in order to better display the visual features, the combination of 3DCNN and LA has been used. This combination will select better features and increase the detection rate.

In the proposed method, 3DCNN-LA is used for feature extraction, selection, and classification. Face images, which are multi-dimensional and RGB, are sent to the pre-trained 3DCNN model to be tuned for emotional face recognition. In this article, the sampling frames of the video file are set to 10 frames. In the first layer, all the inputs are 255 × 255 × 3, and then, in the convolutional layer (CL), it consists of convolutional filters, which are used to extract several local patterns from each local area in the input and generate several feature maps. There are four convolutional layers including Conv1, Conv2, Conv3, and Conv4 in the 3DCNN model, and three of them (Conv1, Conv2, and Conv4) are followed by max-pooling. Convolutional layers use the ReLU activation function. The first convolutional layer is (Conv1) and has 64 cores and a size of 3 × 8 × 8 with a step of 4 pixels and zero-padding. The second one (Conv2) has 256 cores and a size of 64 × 3 × 3 with a step of 1 pixel and a padding of 2. The third convolutional layer (Conv3) has 384 cores with a size of 128 × 3 × 3 connected to the outputs of Conv2, and the fourth convolution layer (Conv4) has 384 cores with a size of 164 × 3 × 3. The ReLU activation function is used to adapt to the training process at the output of each convolutional layer. Based on this, convolution layers are used for extraction and two FC layers for feature selection and finally LA for classification.

To simulate the model SOAR, all goal-oriented symbolic tasks are formulated in problem spaces. A problem space consists of a set of states and operators. States represent situations, and operators refer to actions that, when applied to states, lead to other states. Each functional context contains a goal and roles for a problem state, a state, and an operator. Problem-solving is driven by decisions that lead to selecting problem spaces, states, and operators for the respective context roles. According to a goal, a problem space should be chosen in which the achievement of the goal can be pursued. Then, an initial state must be selected, representing the initial state. Then, an operator must be chosen to apply in the initial state. This process continues until a sequence of operators is discovered, transforming the initial state into a state where the goal is achieved.

SOAR is a cognitive science model, inspired by the functional system of the brain, that implements problem-solving as a goal-directed behavior and involves investigating through the problem and learning from its results. This architecture is used for a wide range of applications, such as routine tasks and solving open-ended problems, which are typical for artificial intelligence and interacting with the outside world, both in simulation and reality (Stavros and Saint, 2010; Laird, 2012). We hypothesize that any brain-inspired model of learning will increase recognition rates. Accordingly, in this article, this model is used to increase the detection rate.

The decision-making process in SOAR is similar to other systems: this process involves matching rules, which is actually a context-dependent representation of knowledge. These conditions describe the current situation of the agent, and the body of rules describes the actions that lead to the creation of structures related to the current conditions in the working memory. SOAR has a deep learning component that deals with procedural knowledge as well as working memory. Reinforcement learning in Soar is relatively simple. This component regulates action selection based on environmental numerical rewards. Numeric priorities specify the expected value of an operator for the current state. When an operator is selected, all rules that determine expected values for this operator are updated based on any new rewards earned by this operator, as well as future expected rewards that may be earned by its successors. This evaluation of operators has been applied to all goals and sub-goals in the system, which enables quick identification of good and bad operators for specific conditions.

In the proposed method, in order to detect emotions, information received from the environment is received and stored by perceptual memory. The initial stages of processing on perceptual memory information are done by procedural memory. With the help of semantic and episodic memories and evaluation stages and the decision process that is done in the short-term working memory, the learning automatons try to learn and repeat the final diagnosis until the final result is produced. The results of the process of learning automata are stored as part of semantic and episodic memories that are long-term memory. The results stored in semantic and episodic memories are returned to the environment as action. All rewards in decision and evaluation processes are stored as working memory operations, and storage of goals and final values is stored as a long-term memory in semantic parts. Improving learning in SOAR model analysis and evaluations can lead to improved final performance.

### 3.1 Improved SOAR model

**Figure 3:**
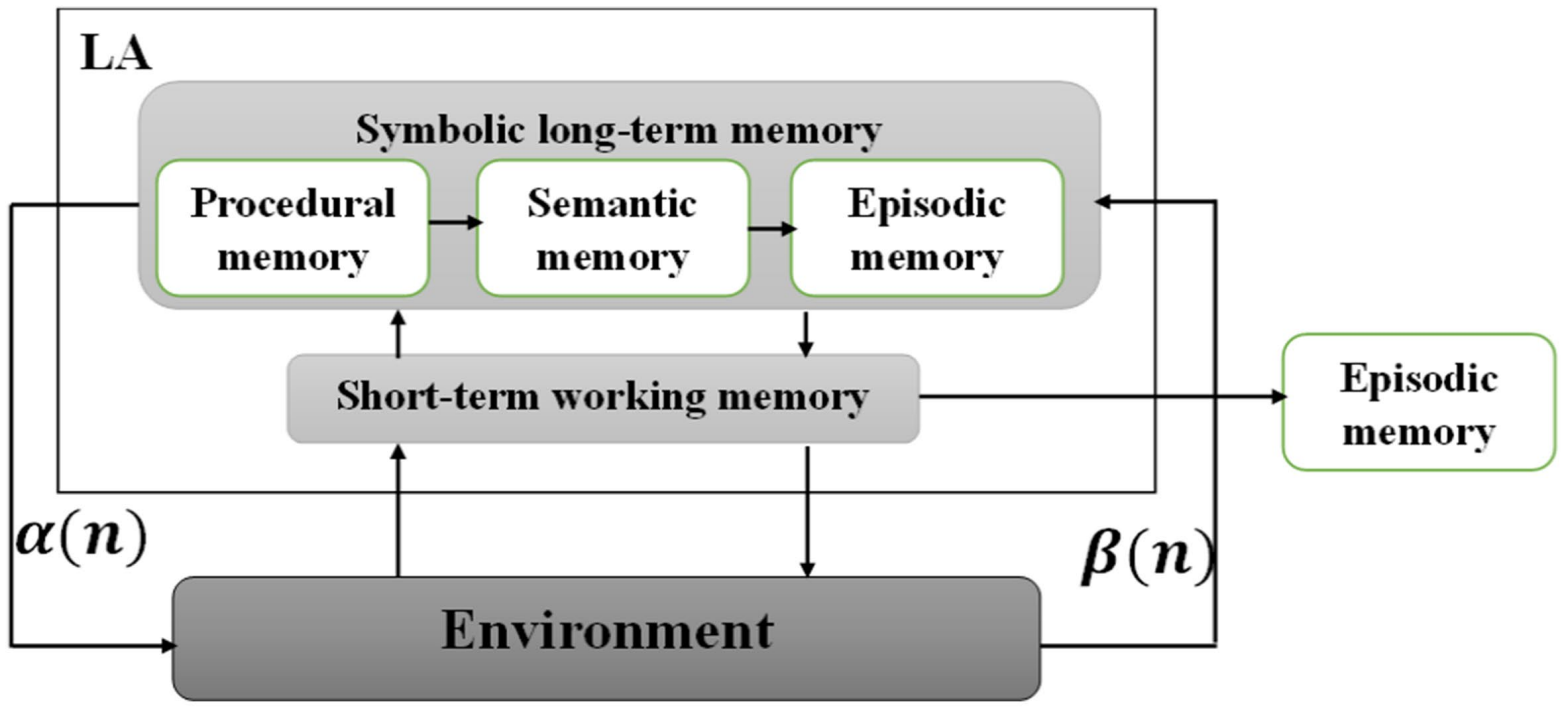
Overview of the improved SOAR model.

The primary model of SOAR includes two main memories, long-term symbolic memory and short-term working memory, which are related to each other. Symbolic long-term memory consists of procedural, semantic, and episodic memory when each performs separate tasks. For example, procedural memory stores previous states of problem-solving, and semantic memory stores known evidence when the recognition related to this memory is done by automatic learning. In addition, information is kept for a while in episodic memory, which contrasts with working memory. The reason for choosing this model of storing and comparing data from long-term to short-term memory is to observe the functioning system of the human brain. The input of the proposed model includes images of facial emotional states. Noise removal and resizing are performed on facial pictures so that the pre-processed inputs enter the proposed model. The memory section will apply image files to all normalized images to extract the best features. All the outputs obtained from the previous step are entered into the symbolic memory, where the best features will be selected. For this purpose, 3DCNN-LA is applied to the features obtained from face images. The training of these networks, including 3DCNN, in calculating the backward propagation error is adjusted by LA to implement the working memory part. In the general description of LA, according to the error rate of 3DCNN, it improves the updating parameter of the neural network weights in the backpropagation algorithm (Figure 3).

Figure 3 shows the improved SOAR model. As explained, the proposed model includes two inputs and each network, with W parameters, and produces class membership probabilities P(C|x,W) C classes according to observation x of emotional state. All network layers, except the softmax layers, had linear unit activation (ReLU) functions (Equation (3)):


(3) f(z)=max(0,z)

We calculated the output of the Softmax layers as follows (Equation (4)):


(4) $$P\left(C|x,W\right)=\frac{\exp\left(z_{C}\right)}{\sum_{q}\exp\left(z_{q}\right)}$$

where $z_{q}$ was the output of neuron q . The outputs of the network were P(C|x,W) . The training process of convolutional deep learning consists of optimizing network parameters W to minimize a cost function for a dataset D . We chose the negative log probability as the cost function (Equation (5)):


(5) $$L\left(W,D\right)=-\frac{1}{\left|D\right|}\overset{\left|D\right|}{\underset{i=0}{\sum }}\log\left(P\left(C^{\left(i\right)}|x^{\left(i\right)},W\right)\right)$$

However, the problem in learning neural networks is that this optimization problem is no longer convex. For this reason, backtracking is one of the standard methods of solving the optimization problem in neural networks. To improve the CNN, the probability theory of the LA in backpropagation error is used to train the improved SOAR model to reduce the complexity of calculations and increase the speed compared with the traditional gradient descent method. The output of LA $\alpha_{i}$ is the possible actions that are used as the momentum factor to update the weights of the neural network. In this way, a strong connection between the best input and output features is created after several iterations. This action adapts the parameters in the gradient descent method to calculate the backpropagation error. The following describes the details of the implementation of the proposed model.

### 3.2 Preprocessing

**Figure 4:**
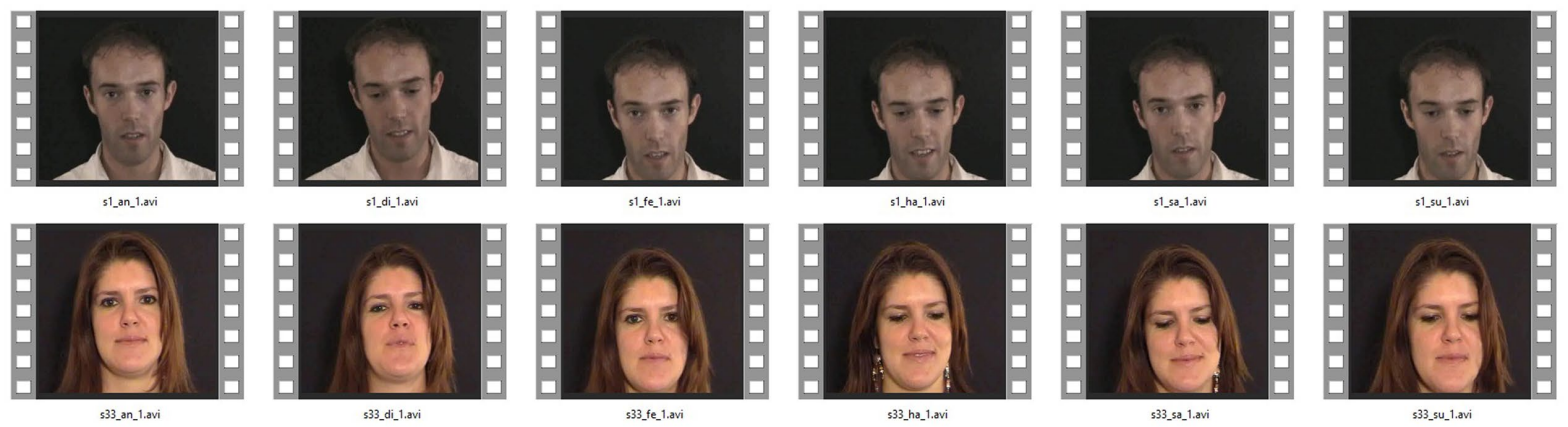
An example of arranged images of different facial expressions in the eNTERFACE’ 05 database. Facial images reproduced from the eNTERFACE’05 EMOTION Database under the terms of the MIT License (https://enterface.net/enterface05/main.php?frame=emotion).

Database preparation and pre-processing operations are performed in the first stage to recognize emotion from facial expressions in the video. These steps are as follows: the database set is divided into two training and testing sections in the database preparation phase. Moreover, 70% of the database set is considered for training and 30% for testing. In the next step, the face image frames are extracted from each video sample and stored in the folders related to video frames. The separation algorithm with a specific frame rate was applied to all the samples to extract the image frames from the video. In these tests, the frame rate of the images is 10 (that is, it extracts 10 frames per second). The average length of eNTERFACE’ 05 database videos is 3 to 4 s. Figure 4 shows examples of arranged images of different facial expressions in the interface 05 databases (Cecchi, 2018).

### 3.3 Feature extraction, feature selection, and classification

In the proposed method, in the procedural memory part of the improved SOAR model, all images are pre-processed and segmented. Then, 3DCNN-LA will be applied to the classification from facial expressions. The reason for choosing 3DCNN on face images is that no dimension is removed from the images, and considering the temporal information in dynamic images leads to more efficient and better feature selection. The training of 3DCNN networks in calculating the backward propagation error is adjusted by LA so that the working memory part can be implemented. Finally, the features obtained in the decision-making stage will be fused to obtain a recognition rate based on visual information. In this article, 3DCNN has been used to use more features of images in video, such as in studies (Liu and Durlofsky, 2021; Shinde and Patidar, 2023). Dimensional reduction simplifies the existing variables to be fewer without losing the information contained in the initial data.

CNN or ConvNet is a deep feed-forward artificial neural network, which is widely applied in image analysis. CNN consists of an input layer (input layer), an output layer (output layer), and several hidden layers (hidden layer). Hidden layers generally contain convolutional layers, pooling layers, normalization layers, ReLU layers, total connected layers, and loss layers. All the layers are arranged in a pile. CNN uses a three-dimensional architecture, namely, width, height, and depth. The width and height dimensions on CNN are representations of the image (texture and morphology), while the inner dimensions represent color channels.

## 4 Results and experiments

All the tests of this article are implemented by MATLAB 2022 software, and the accuracy test is done using the K-Fold method with *K* = 10. The measurement criterion of each part is recognition confusion matrices (Saxena et al., 2022). In the image section, a sequence of 10 frames of colored face images, each with a size of 100×96, is fed to the 3DCNN-LA neural network. In the generated convolutional neural networks 3DCNN-LA, the size of each batch is 32, and the number of repetition steps is 500. The learning rate is 0.001, and the momentum rate is 0.0001. In this method, the early stopping technique is used during training.

### 4.1 Recognition of facial emotional states

Table 1 shows the confusion matrix of recognition rate of facial emotional states using improved SOAR on the eNTERFACE’ 05 database.

**Table 1:** Confusion matrix of recognition of visual emotional states using improved SOAR.

	Anger	Disgust	Fear	Happiness	Sadness	Surprise
Anger	85%	2%	3%	2%	3%	5%
Disgust	3%	89%	3%	1%	2%	2%
Fear	2%	3%	80%	6%	7%	2%
Happiness	3%	2%	3%	88%	2%	2%
Sadness	2%	4%	2%	2%	87%	3%
Surprise	2%	4%	3%	5%	3%	83%

As shown in Table 1, the recognition rate of facial emotions using the improved SOAR model is entirely different. The results revealed that “disgust” has the highest recognition rate and “fear” has the lowest recognition rate, with values of 89 and 80%, respectively. The reason for such results can also be that the above recognizable cases are mostly evident emotions, and these cases are visible in the face, such as disgust, which has a high percentage detection rate. However, an emotion such as fear, which has the lowest value, is not visible in the face, and many people can hide this emotion behind their faces (Liu and Durlofsky, 2021). The details of the proposed method are shown in Figure 3. This causes the separate benefit of each classification and the final improvement to produce better answers. For integration at the decision level, various methods of combination rules have been tested, including minimum, maximum, average, product, and combination. Each combination of the above regulations is tested for integration at the decision level in the proposed model, and the performance results of each are shown in Table 1. The next stage of the experiments is to apply the proposed model without considering the phase of extracting the features of the evidence. Table 2 shows the confusion matrix of the proposed model to recognize facial emotional states on six basic emotion classes from the eNTERFACE ‘05 database.

**Table 2:** Confusion matrix of recognition of facial emotional states using SOAR model.

	Anger	Disgust	Fear	Happiness	Sadness	Surprise
Anger	77%	3%	7%	3%	4%	6%
Disgust	1%	91%	3%	1%	2%	2%
Fear	1%	4%	81%	5%	7%	2%
Happiness	2%	3%	2%	90%	2%	1%
Sadness	2%	4%	2%	4%	87%	1%
Surprise	2%	4%	6%	2%	2%	84%

Table 2 shows that the emotional state of “disgust” and “anger” has the highest and lowest recognition rates, respectively, in facial emotional state recognition. By examining the results of the proposed model, it was found that the feeling of disgust has the highest detection rate in the detection of facial emotional states. The visual characteristics have entirely changed according to the examination carried out in this emotional state. They can be observed when this action causes a better diagnosis of this case. The proposed method is used to achieve more feature depth than an additional step in network iterations to increase the recognition rate and take advantage of LA. Table 3 compares the proposed method with 3DCNN, 3DCNN-LA, and improved 3DCNN-LA. All the tests of this article are implemented, and the accuracy test is done by K-Fold cross-validation (*K* = 10; Pandian, 2022).

**Table 3:** Comparison of the proposed method in different cases.

Row	Method	Accuracy (%)
1	CNN	81.4
2	3DCNN	83.27
3	3DCNN-LA	85.3

According to Table 3, the proposed method using CNN has achieved an accuracy of 81.4%. When 3DCNN is used to improve this method, a value of 27.83 is obtained. A 3D convolutional neural network is based on the concept of convolutional neural network (CNN) but with the addition of a time dimension. In a traditional 2D CNN, the input consists of several image frames that the network processes and analyzes. Adding the time dimension improves the results, which, according to this table, has achieved an improvement of 1.87%. Time dimension is very important in video files and can be effective in detection. Finally, the proposed 3DCNN-LA method, which in addition to using the advantages of the time dimension of 3DCNN, uses LA as a learning reward calculator in FC layers, has reached a value of 85.3% in the accuracy criterion. In the next evaluation, we will compare and check the proposed method with similar methods. In CNN and 3DCNN, by adding another FC layer, the classifier value was obtained, but in the proposed method, the second FC is done using LA.

As shown in Table 4, the proposed method has shown a better value in the accuracy criterion than similar methods and has recorded 4.2% improvement with the closest method in terms of this criterion. The closest method to the method proposed in this article was the method presented in the study mentioned in Liang et al. (2020), which also jointly models the spatial characteristics and temporal dynamics using a deep convolution network for FER. Nevertheless, the proposed method can achieve better accuracy by increasing the work on feature selection and extraction. However, preprocessing and feature selection take more time.

**Table 4:** Comparison of recent models.

Method	Accuracy (%)
Song (2021)	74.5
Huang et al. (2023)	77.37
Li and Gao (2022)	77.92
Arora et al. (2022)	79.98
Li et al. (2018)	80.54
Liang et al. (2020)	81.1
Ours	**85.3**

## 5 Conclusion

With the increase in the interaction between machine and human and the need to recognize human emotions to advance industry goals, discovering the emotional states of human faces plays a fundamental role. According to studies, it is not reliable to recognize human emotions by facial expression alone. As a result, several methods should complement each other to increase the detection performance. In this article, the proposed method uses improved SOAR to detect emotions from facial expressions. Our improved SOAR model, based on 3DCNN-LA, is especially designed to consider both the extracted frames’ features and their sequences. Meanwhile, LA helps fine-tune the weights, making them more targeted than previous detection models. 3DCNN processes the sampled point data of the image; they take the raw pixel data as input, train the designed architecture, and automatically extract the features. Our proposed model includes several main objectives. The first goal is to provide a model to learn the time order of frames in the movie, and the second goal is to provide a model to better display the visual features and finally increase the recognition rate. These models do not consider the spatiotemporal parameters that occur from the consecutive frames of a video recording. At the same time, the improved SOAR based on 3DCNN-LA processes the sequence data and the images themselves. This allows our model to produce a smaller, more accurate, and computationally efficient model. The combination of 3DCNN and LA in improved SOAR utilizes richer information in extracting image features, which leads to better image description, thus improving detection and target recognition performance. The classification of the proposed method using an improved SOAR model with a combination of 3DCNN and LA produces the output. It provides the recognition rate of the expression of the calculated visual emotional states. The highest and lowest rates of recognition based on facial expressions were related to expressions of hatred at 89% and fear at 80%, respectively. The proposed model has recorded an average improvement of 4.2% compared with the closest model. According to the computational volume of the proposed method, this method can increase the speed of emotion recognition by using supercomputers.

## Data availability statement

The original contributions presented in the study are included in the article/supplementary material, further inquiries can be directed to the corresponding author.

## Ethics statement

The studies involving humans were approved by the Department of Computer Engineering, Rasht Branch, Islamic Azad University, Rasht, Iran. The studies were conducted in accordance with the local legislation and institutional requirements. The participants provided their written informed consent to participate in this study.

## Author contributions

MR: Writing – original draft, Writing – review & editing. SM: Writing – original draft, Writing – review & editing. MY: Writing – original draft, Writing – review & editing.
